# Early Operation in Perforating Gastric Ulcer

**Published:** 1901-03-30

**Authors:** 


### EARLY OPERATION IN PERFORATING GASTRIC ULCER.

On every side the same note is being sounded in regard to the necessity for early diagnosis as a means of giving opportunity for early operative treatment in all kinds of abdominal disease. Just now surgeons are specially directing their attention to diseases of the stomach, and every week in almost every journal, and on both sides of the Atlantic, cases are being recorded which go to show how much surgery can do in these disorders. A few weeks ago we referred to the experience of Mr. Mayo Robson in regard to the surgical treatment of bleeding ulcers of the stomach; now we would call attention to the urgent plea put forward by Dr. Maunsell,[1] of Dublin, for early diagnosis and early treatment in cases of perforated gastric ulcer. He points out that we must, in the light of more recent knowledge, look with extreme suspicion on the signs and symptoms of gastric ulcer and perforation as given in the standard text-books, for the previous history in some of these cases would have hardly satisfied physicians of a few years ago that even gastralgia was present. There is always, however, some symptom pointing in an indefinite manner to a gastric source. The onset of the symptom of perforation is sudden, and frequently takes place during some exertion. But although severe the onset may not incapacitate, for the patient may be able to walk to hospital. The points in regard to diagnosis to which he draws special attention are (1) the pain, which begins in the epigastrium, spreads, but does not shift its position; (2) there is no pain on micturition, which is a frequent sign in peritonitis of the lower abdomen; (3) thirst is not intense, and there is no flinging about as in hæmorrhage; (4) there may be no distention in muscular subjects, the tympanites and free gas being only shown by the encroachment on the thoracic area; (5) the pulse cannot be relied upon in forming an early diagnosis; (6) the statement sometimes made that a "stomach note" excludes perforation is unwarrantable, as a perforated and collapsed stomach are by no means synonymous terms; (7) liver dulness is diminished or absent in almost every case.

[1] Brit. Med. Journ., March 23.

### TUBERCULOUS PERITONITIS.

Speaking recently at the Polyclinic, Dr. Burney Yeo[1] drew attention to a form of medical treatment which he has found useful in cases of tuberculous peritonitis. As he justly says, much of the remarkable improvement in the prognosis of this disease which has taken place during late years is due to the progress which has been made in abdominal surgery, but he holds that progress has also been made in the medical measures which are at our disposal. The measures to which he more particularly draws attention in his lecture are the external application of a mixture of equal parts of iodoform ointment and cod-liver oil, which is to be rubbed in freely over the abdominal surface twice a day, and the administration of a pill containing a quarter of a grain of iodoform and half a minim of creasote thrice daily. He gives three illustrative cases which he thinks worthy of attention, in that they are not picked cases but occurred in close succession within a few months of one another, were all treated in the same way, and all ended in complete recovery. As to the *rationale* of the proceeding, Dr. Yeo points to the fact that we remain very much in the dark even as regards the *rationale* of the surgical proceedings, which are admitted on all hands to be successful. He thinks, however, that the suggestion made by Mr. Watson Cheyne is a reasonable one—viz., that after the removal of the fluid from the peritoneal cavity by incision, serum having anti-bacterial properties may be poured out, and the morbid process may thus be arrested. The important fact is that a very slight change in the environment of a pathogenic organism is sufficient to arrest or nullify its activities and to lead to its disappearance, and Dr. Yeo holds that when iodoform is rubbed into the abdomen in a young person it probably rapidly enters the blood and is, if regularly applied, continuously eliminated in the secretions, including the secretions into the serous cavities, which must in time become pretty richly charged with iodine compounds, at any rate sufficiently so to act as antitoxin to the tubercle toxin, or as anti-bacterial to the tubercle bacilli. As furnishing support to the idea that iodine compounds do possess some "anti" power, he refers to the general idea that iodine is an antitoxin to tubercle, which has long existed in the minds of physicians. Antitoxins, using the word in its wider sense, are not limited he says to the animal products, the oldest and surest antitoxins which we possess being mercury and quinine.

[1] Lancet, March 16.

